# Possible cross-feeding pathway of facultative methylotroph *Methyloceanibacter caenitepidi* Gela4 on methanotroph *Methylocaldum marinum* S8

**DOI:** 10.1371/journal.pone.0213535

**Published:** 2019-03-14

**Authors:** Mio Takeuchi, Haruka Ozaki, Satoshi Hiraoka, Yoichi Kamagata, Susumu Sakata, Hideyoshi Yoshioka, Wataru Iwasaki

**Affiliations:** 1 Institute for Geo-resources and Environments, National Institute of Advanced Industrial Science and Technology (AIST), Higashi, Tsukuba, Ibaraki, Japan; 2 Department of Bioinformatics, Faculty of Medicine, University of Tsukuba, Tennodai, Tsukuba, Japan; 3 Department of Computational Biology and Medical Sciences, Graduate School of Frontier Sciences, the University of Tokyo, Kashiwa-no-ha, Kashiwa, Chiba, Japan; 4 Bioproduction Research Institute, AIST, Higashi, Tsukuba, Ibaraki, Japan; 5 Department of Biological Sciences, Graduate School of Science, the University of Tokyo, Yayoi, Bunkyo-ku, Tokyo, Japan; University of Münster, GERMANY

## Abstract

Non-methanotrophic bacteria such as methylotrophs often coexist with methane-oxidizing bacteria (methanotrophs) by cross-feeding on methane-derived carbon. Methanol has long been considered a major compound that mediates cross-feeding of methane-derived carbon. Despite the potential importance of cross-feeding in the global carbon cycle, only a few studies have actually explored metabolic responses of a bacteria when cross-feeding on a methanotroph. Recently, we isolated a novel facultative methylotroph, *Methyloceanibacter caenitepidi* Gela4, which grows syntrophically with the methanotroph, *Methylocaldum marinum* S8. To assess the potential metabolic pathways in *M*. *caenitepidi* Gela4 co-cultured with *M*. *marinum* S8, we conducted genomic analyses of the two strains, as well as RNA-Seq and chemical analyses of *M*. *caenitepidi* Gela4, both in pure culture with methanol and in co-culture with methanotrophs. Genes involved in the serine pathway were downregulated in *M*. *caenitepidi* Gela4 under co-culture conditions, and methanol was below the detection limit (< 310 nM) in both pure culture of *M*. *marinum* S8 and co-culture. In contrast, genes involved in the tricarboxylic acid cycle, as well as acetyl-CoA synthetase, were upregulated in *M*. *caenitepidi* Gela4 under co-culture conditions. Notably, a pure culture of *M*. *marinum* S8 produced acetate (< 16 μM) during growth. These results suggested that an organic compound other than methanol, possibly acetate, might be the major carbon source for *M*. *caenitepidi* Gela4 cross-fed by *M*. *marinum* S8. Co-culture of *M*. *caenitepidi* Gela4 and *M*. *marinum* S8 may represent a model system to further study methanol-independent cross-feeding from methanotrophs to non-methanotrophic bacteria.

## Introduction

Microbial methane oxidation plays an important role in the global methane cycle. Aerobic methane-oxidizing bacteria (methanotrophs) are key players in aerobic and micro-aerobic environments. The development of stable isotope probing has highlighted that methane-derived carbon is incorporated not only by methanotrophs but also by non-methanotrophic bacteria (methylotrophs or others) in diverse environments, and suggests the global importance of cross-feeding interactions in methane oxidation [1, 2, 3, 4]. Methanol is synthesized in the periplasm of methanotrophs, and therefore, may diffuse out of the cell [5]. Hence, it is generally speculated that the co-existence of methanotrophs and non-methanotrophic bacteria mainly involves the cross-feeding of methanol excreted by methanotrophs to non-methanotrophic bacteria [2]. Methanol-dependent cross-feeding between methanotrophs and non-methanotrophic bacteria has been studied since the 1970s [6, 7]. Using isolates from lake sediments, Krause et al. [8] demonstrated that methanol excreted by a methanotroph is indeed the sole carbon and energy source for the co-existing obligate methylotroph that can utilize methanol or methylamine exclusively.

On the other hand, some methanotrophs are also known to excrete some organic acids [9]. It is speculated that these organic acids are derived from dead cells. Recently, Kalyuzhnaya et al. [10] demonstrated that *Methylomicrobium alcaliphilum* of Gammaproteobacteria release organic compounds, such as formate and acetate, via fermentation-based methanotrophy. This suggested that cross-feeding of methane-derived carbon from a methanotroph to non-methanotrophic bacteria may not be mediated solely by methanol and may involve more diverse processes. However, this has not been extensively explored yet.

Recently, we obtained a methane-oxidizing enrichment culture from marine sediments of Kagoshima Bay, Japan and isolated a novel facultative methylotroph, *Methyloceanibacter caenitepidi* Gela4 [11] and a novel gammaproteobacterial methanotroph, *Methylocaldum marinum* S8 [12]. *M*. *caenitepidi* Gela4 grows on a wide range of substrates and can grow syntrophically with *M*. *marinum* S8 using methane added as the sole carbon source. The co-culture of *M*. *caenitepidi* Gela4 and *M*. *marinum* S8 would be a suitable model system for addressing the nature of a facultative methylotroph cross-feeding on a methanotroph.

In this study, we aimed to reveal the metabolic pathways employed by a marine facultative methylotroph cross-feeding on a methanotroph using genome analysis, RNA sequencing (RNA-Seq), and chemical analysis.

## Materials and methods

### Bacterial strains and culture conditions

*M*. *caenitepidi* Gela4 was grown in NMS-SP medium [12] with 1% (v/v) methanol added as a carbon source at 36°C. *M*. *marinum* S8 was grown in NMS-SP medium with a headspace of methane and air (20:80, v/v). For the co-culture of *M*. *caenitepidi* Gela4 and *M*. *marinum* S8, each culture was harvested by centrifugation (4,200 × *g* for 20 min), washed twice with NMS-SP medium and grown together in NMS-SP medium of 40 mL at 36°C in a 200 mL glass bottle under methane/air (20:80, v/v). Culture growth was monitored by measuring the optical density at 660 nm. Cells were harvested by centrifugation at 4,200 × *g* for 20 min. For DNA analysis, the cells were then stored at −20°C. For RNA analysis, the supernatant was discarded after centrifugation, and 1.5 mL of RNAlater (Ambion Inc., Austin, TX, USA) was added. Re-suspended cells were stored in a refrigerator overnight. The cells were then harvested and stored at −80°C before RNA extraction, as described below.

### Cell counts

*M*. *caenitepidi* Gela4 and *M*. *marinum* S8 cells are morphologically distinct as visualized by a microscope [11, 12]. Therefore, growth of *M*. *caenitepidi* Gela4 and *M*. *marinum* S8 in co-culture was monitored by cell count via microscopy. Cells were taken periodically and counted by a haemocytometer.

### Genome sequencing, assembly, and annotation

Chromosomal DNA was extracted using an ArchivePure DNA tissue kit (5 Prime, Gaithersburg, MD, USA) according to the manufacturer’s instructions. The draft genome sequence of *M*. *caenitepidi* Gela4 comprising of two scaffolds was generated previously [11]. The remaining gap was closed by primer-walking, using primers complementary to the contig ends, at Takara Bio Inc. (Shiga, Japan). Whole-genome shotgun sequencing of *M*. *marinum* S8 was performed using PacBio RS II (Pacific Biosciences, Menlo Park, CA, USA) according to the manufacturer’s protocol. Genomes were assembled into a single contig using Sprai pipeline version 0.9.9.19 (http://zombie.cb.k.u-tokyo.ac.jp/sprai/) and manual curation. The contig was polished with Quiver [13].

The Microbial Genome Annotation Pipeline (MiGAP) was used to annotate sequences [14]. Then, gene names were then assigned to genome annotations based on the best hits of blastp searches (version 2.2.30+; E-value<1e-5) [15] against the SwissProt database (2017_2), with several genes annotated manually. The percentage amino acid identities between the predicted sequences determined in this study were calculated using the pairwise distances program in MEGA version 7 [16]. The Circos package [17] was used to generate Circular plots. Genes were assigned to Kyoto Encyclopedia of Genes and Genomes **(**KEGG) pathway modules using metabolic and physiological potential evaluator (MAPLE) [18]. Only modules with at least three assigned genes and qValue < 0.1 were retained, leading to 92 KEGG pathway modules for *M*. *caenitepidi* Gela4 (S1 Table) and 100 modules for *M*. *marinum* S8 (S2 Table).

The in-house Julia scripts were used to define the duplicated genes in each species. The Bio.jl Julia package (https://github.com/BioJulia/Bio.jl) was used to translate the annotated CDS in each genome into amino acid sequences. All-against-all blastp search was performed using the translated amino acid sequences and blast+ (version 2.2.30+) (E-value < 1e-5). A duplicated gene network, with nodes representing gene products and edges representing pairs of gene products, with E-value < 1e-5 and > 95% identity, was constructed. Genes with at least one edge in the duplicated gene network were defined as duplicated genes.

### RNA extraction and sequencing

RNA-Seq was used to compare the transcriptome profiles of *M*. *caenitepidi* Gela4 grown in pure culture on methanol with cells grown in co-culture with *M*. *marinum* S8 on methane-supplemented medium. Two replicate cultures at late exponential phase were prepared for each culture condition. The NucleoSpin RNA kit (Macherey-Nagel, Düren, Germany) was used to extract total RNA. Subsequent sample processing, including rRNA subtraction, library prep, and sequencing, was performed at Takara Bio Inc. Ribosomal RNA was removed from total RNA using the Ribo-Zero rRNA removal kit (Epicenter Biotechnologies, Madison, WI, USA). An Agilent 2100 Bioanalyzer was used to verify sample quality. RNA-Seq libraries were created using the Illumina TruSeq RNA sample prep kit version 2. All cDNA samples were individually barcoded and sequenced in the same lane on the HiSeq 2500 platform. The number of cDNA sequencing reads generated per sample varied between 22.0 and 29.9 million.

### Bioinformatics analysis of the transcriptome data

For mapping of RNA-Seq reads, the genome sequences of the two species were combined into two chromosomes in a FASTA file. RNA-Seq data were mapped to the genome by STAR (version 2.5.2b) [19] with the parameters “—outFilterMismatchNmax 3—outFilterMultimapNmax 10—outSAMprimaryFlag AllBestScore—outSAMstrandField None—alignIntronMax 3—alignIntronMin 4”. BAM files were processed using samtools 1.3 [20]. Specifically, uniquely mapped reads were selected with the parameter “-q 255” and primary alignment reads were selected with the parameter “-F 256”. The featureCounts option in the subread package (version 1.5.1) [21] was used to count the number of reads mapped to each gene. For stain S8 data, the parameters “-M–fraction” were also set to calculate fractional counts for multi-mapping reads. Using simulated RNA-Seq data composed of the sequencing reads generated from the complete genome sequences of the two species, we confirmed that the aforementioned data processing strategy enabled unambiguous separation of the sequencing reads into two species (S3 and S4 Tables). Specifically, using custom Julia scripts, we generated simulated RNA-Seq data of each strain as FASTA files by extracting 100-bp length sequences of regions with at least 1-bp overlaps with genes. The sequences were prepared for each region from both forward and reverse strands. Reads were mapped to the combined genome of two strains and the primary alignment reads were selected as described above. Then, reads were counted for each CDS using the FeatureCounts with the parameters "-M—fraction". Differentially expressed genes were identified using edgeR (version 3.16.5) [22] by quasi-likelihood test with genewise negative binomial generalized linear models and analysed by multivariate analysis. Only genes with ≥ 10 raw read counts in at least one sample were considered for the differentially expressed gene analysis. The reads per million mapped reads (RPM) value was calculated for each gene by dividing the read count by the total number of mapped reads. The reads per kb of transcript per million mapped reads (RPKM) value was then calculated for each gene, by dividing the RPM value by CDS length (unit = kb). The data for *M*. *caenitepidi* Gela4 were based on unique mapping analysis, while the data for *M*. *marinum* S8 were based on a primary alignment because of extensive gene duplication in the genome.

### Chemical analysis

Triplicate samples for chemical analysis were taken at the beginning of the incubation period and at early and late exponential phase. Samples of 2 mL were withdrawn using a syringe and filtered through a 0.22-μm pore size filter. Samples for methanol and acetaldehyde analysis were immediately sealed in a glass ampoule. Samples for organic acids and ammonium were stored in a plastic tube. All of the samples were stored at −20°C before analysis. Methanol and acetaldehyde concentrations were analysed by the Agilent 7697A Headspace Sampler coupled to the Agilent 6890N Gas Chromatograph/MSD system with selected ion monitoring mode. One millilitre of the sample was added to a 5 mL headspace vial, heated at 80°C for 5 min, and 3 mL of headspace vapour was injected into a split injector of the gas chromatograph. The DB-WAX capillary column (60 m × 0.25 mm × 0.50 μm, Agilent J&W Scientific, CA, USA) was used as a GC column. Helium was the carrier gas at a flow rate of 1.0 mL/min. The detection limits for methanol and acetaldehyde were 310 and 570 nmol/L, respectively. Concentrations of organic acids were analysed via HPLC (Shimadzu Corp., Japan) with a conductivity detector. Shim-pack SCR-102H (300 mm × 8 mm, Shimadzu model, Shimadzu Corp., Japan) was used as a column to separate organic acids eluted in 5 mmol/L of p-toluenesulfonic acid at 40°C. The detection limits were 1 μmol/L for formate and acetate and 10 μmol/L for propionate and butyrate.

### Real-time quantitative reverse transcription PCR

To support the RNA-Seq analysis results, expression levels of acetyl-CoA synthetase gene (*acsA*), hydroxypyruvate reductase gene (*hpr*), and aldehyde dehydrogenase gene (*aldA*) were quantified by real-time quantitative reverse transcription PCR assay. Co-cultures was incubated in triplicate and harvested at early and late exponential phases. *M*. *caenitepidi* Gela4 cells grown with methanol or sodium acetate (0.3%) were collected at late exponential phase and also analysed as reference. Total RNA was extracted as described above. For cDNA synthesis, 400–500 ng of RNA was reverse-transcribed using the PrimeScript RT reagent kit (Takara Bio, Japan) according to the manufacturer’s instruction. Primers specific for *acsA*, *hpr* and *aldA* genes of *M*. *caenitepidi* Gela4 were designed using Primer3 [23]. The primers for *acsA* gene were GelaACS-F (5ʹ - GAACTCGACCTTTGCGTATCCG-3ʹ) and GelaACS-R (5ʹ - TGCAGTTCGCCGACACGTTAAG-3ʹ). The primers for *hpr* gene were GelaHPR-F (5ʹ - GATGCCGAACTTCATCGTCACG-3ʹ) and GelaHPR-R (5ʹ - GTTGCCCGCAACGAACGCATC-3ʹ). The primers for *aldA* gene were Gald-F (5ʹ - CGACACCGTTGCCTATCATTTC-3ʹ) and Gald-R (5ʹ - TCCAGCACGCCATCAAAATC-3ʹ). PCR amplification was not detected with these three primer sets when *M*. *marinum* S8 genomic DNA was used as a template. Real-time PCR was performed with TB Green Premix Ex Taq II (Takara Bio, Japan) using a Thermal Cycler Dice Real Time System II (Takara Bio, Japan). Each real-time PCR was carried out in a 15-μl total volume reactions that contained cDNA (1μl), Mastermix, forward and reverse primers (0.4 μM each) and nuclease-free sterile water. The thermal cycler program consisted of 40 cycles of denaturation (95°C for 5s) and annealing and extension (55°C for 30s) after an initial denaturation at 95°C for 30s. To construct calibration standard curves, dilution series of positive control DNA were used. For positive control DNA, gene fragments were amplified with the primers described above from *M*. *caenitepidi* Gela4 genomic DNA. PCR products were then separated by gel electrophoresis and extracted with a NucleoSpin Gel and PCR Cleanup kit (Macherey-Nagel, Düren, Germany).

## Results

### Complete genome of *M*. *caenitepidi* Gela4 and possible metabolic pathways

**Fig 1:**
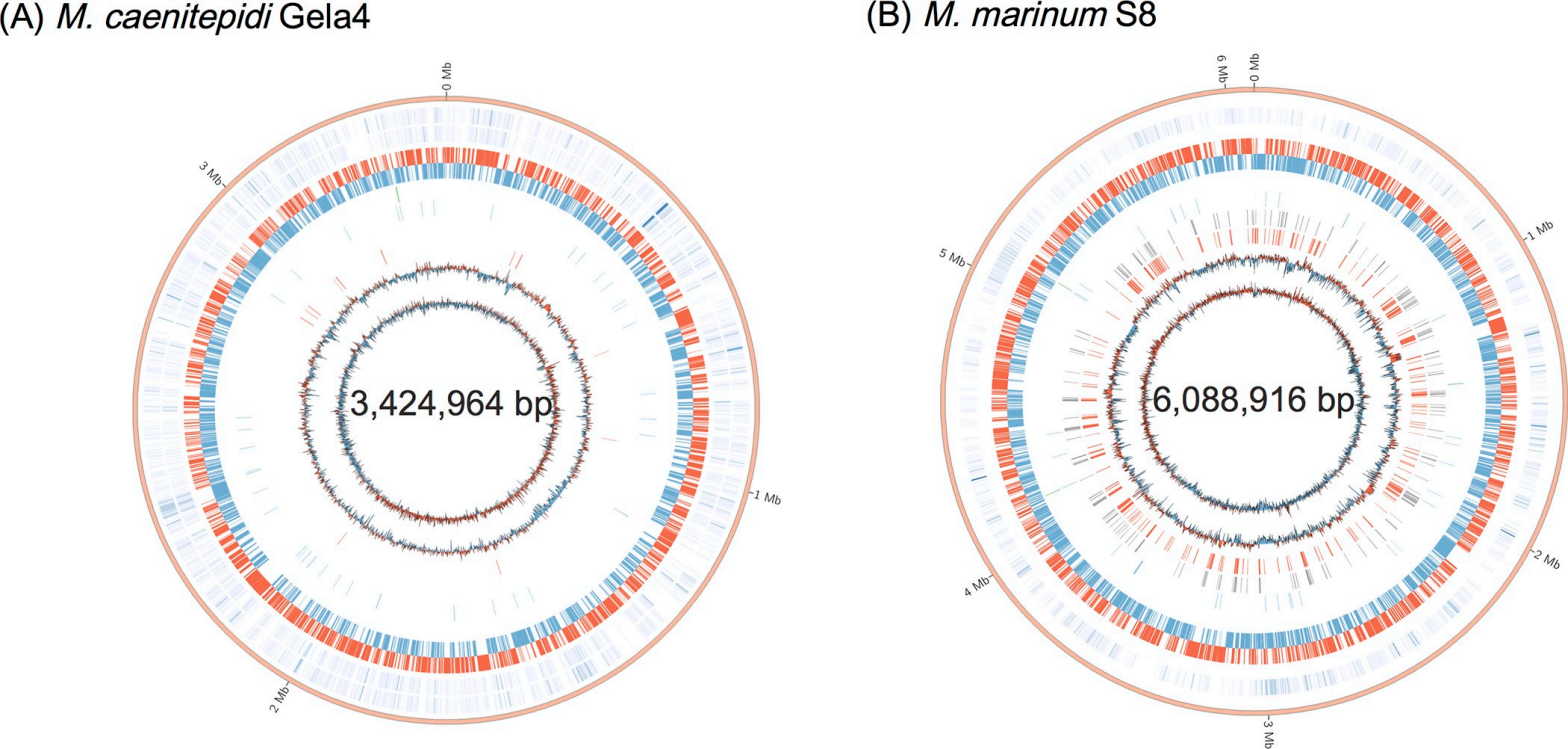
**Circular diagrams of the (A) *M*. *caenitepidi* Gela4 and (B) *M*. *marinum* S8 genomes.** Circles (outside to inside) depict (1) contigs; (2) gene expression levels in pure culture, log10 (RPKM + 1) (only for *M*. *caenitepidi* Gela4); (3) gene expression levels in co-culture, log10 (RPKM + 1); (4) coding genes on the leading strand (red); (5) coding genes on the lagging strand (blue); (6) 16S rRNA genes (green); (7) tRNA genes (blue); (8) transposase genes (grey); (9) duplicated genes (E-value<1e-5, % identity >95.0) (red); (10) the GC content (1-kb sliding window); and (11) the GC skew (1-kb sliding window).

We generated the complete genome of *M*. *caenitepidi* Gela4 by closing the gaps from the previously published draft genome [9]. The *M*. *caenitepidi* Gela4 genome was obtained at a sequencing coverage of 76×. It consisted of a single circular chromosome of 3,424,964 bp, with a G+C content of 62.8% (Fig 1A). The genome was found to contain one ribosomal RNA operon (16S–23S–5S), 45 tRNA genes and 3,342 coding sequences (CDSs) (S5 Table).

In *M*. *caenitepidi* Gela4, pathways for oxidization of methanol to formate were identified. The gene clusters responsible for methanol oxidation (*mxaFJGIRSACKLD*, GL4_0421–0431) and the synthesis of the cofactor pyrroloquinoline quinone (PQQ; *pqqBCDE*) were identified (GL4_3155, 2349–2351). In addition, four genes encoding a methanol dehydrogenase large subunit similar to *xoxF* were found (GL4_0437, 1130, 1360, and 1361). The amino acid sequences of four XoxF proteins shared 70%–94% identity with each other. All genes coding for enzymes involved in tetrahydromethanopterin (H_4_MPT)-mediated (*fae*, *mtdB*, *mch* and *fhcD*; GL4_1783 and 3256, 1788, 1786, 1794) and tetrahydrofolate (H_4_F)-mediated (*mtdA*, *fch* and *ftfL*; GL4_3376, 3375 and 3380) formaldehyde oxidation were identified. Genes for two formate dehydrogenases (FDH), NAD-dependent-FDH (*fdhA1*, *B1*, *C1*, *D1*, and *E1*; GL4_0089, 1367–1370), and tungsten-containing FDH (*fdhA2* and *B2*; GL4_3220 and 3221) were also identified.

Methylotrophs assimilate carbon from methanol by using either the ribulose monophosphate (RuMP) pathway or the serine cycle [24]. In *M*. *caenitepidi* Gela4, all genes necessary for the serine pathway, i.e. serine hydroxymethyltransferase (*glyA*; GL4_1969), serine glyoxylate aminotransferase (*sga*; GL4_3378), hydroxypyruvate reductase (*hpr*; GL4_3377), d-glycerate 2-kinase (*gckA*; GL4_3381), enolase (*eno*; GL4_2130), phosphoenolpyruvate carboxylase (*ppc*; GL4_3372), malate dehydrogenase (*mdh*; GL4_0105), malate-CoA ligase (*mtk*; GL4_3373 and 3374) and malyl-CoA lyase (*mcl*; GL4_3371) were identified. In contrast, genes for the RuMP pathway, i.e. 3-hexulose-6-P synthase (*hps*) and 6-phospho-3-hexuloisomerase (*phi*), were not detected. The gene for isocitrate lyase was not found, but genes for the ethylmalonyl-CoA (EMC) pathway were identified as a glyoxylate regeneration pathway. These gene composition patterns indicated that *M*. *caenitepidi* Gela4 assimilates carbon from methanol through the serine pathway. Moreover, genes for glycolysis and the tricarboxylic acid (TCA) cycle were also identified.

### Complete genome of *M*. *marinum* S8 and potential metabolic pathways

We generated the complete genome of *M*. *marinum* S8 obtained at 102 × sequencing coverage using PacBio RS II. The genome was found to consist of a single circular chromosome of 6,088,916 bp, with a G + C content of 58.7% (Fig 1B). The genome contains 48 tRNA genes and 5,611 CDSs (Fig 1B and S6 Table). Notably, it possessed a high number of transposase genes and duplicated genes (Fig 1B); 9.5% (539) of genes were identified as duplicate when the threshold of amino acid sequence identity was set at >95% (S1 Fig, S7 Table). In addition, the genome contains two ribosomal operons (16S–23S–5S).

Among the duplicated genes were key genes involved in methane oxidization. Two sets of particulate methane monooxygenase operons (*pmoABC*; sS8_1787–1789 and 3975–3977) were found; *pmoA* and *pmoC* genes were identical, whereas the predicted *pmoB* gene products showed 99.5% amino acid identity. In addition, the genome was found to contain three additional *pmoC* genes (sS8_2529, 3418 and 4876), whose predicted products shared a 60%–91% amino acid identity with the PmoC protein encoded by the *pmo* operon.

Four genes predicted to encode the large subunit of methanol dehydrogenase were also found. One of them is located in a gene cluster encoding methanol dehydrogenase (MDH) and accessory proteins (*mxaFJGIRSACKLD*; sS8_1426–1436). The other three are similar to *xoxF* (sS8_0935, 1979 and 2523). Four genes for PQQ biosynthesis were found in the same cluster (*pqqBCDE*; sS8_2216–2219). All genes coding for enzymes involved in H_4_MPT-mediated and H_4_F-mediated formaldehyde oxidation were also identified. *M*. *marinum* S8 genome contains genes encoding the complete RuMP pathway, i.e. *hps* (sS8_1135 and 1148) and *phi* (sS8_1134) (S6 and S8 Tables). For the cleavage part of the RuMP pathway, genes for both the Entner-Doudoroff pathway (sS8_4013, 4180, 4181, 4179, 1800) and Embden-Meyerhof-Parmas pathway (sS8_4382, 1130) were identified. Among genes for the serine pathway, *M*. *marinum* S8 genome contains serine hydroxymethyltransferase (sS8_0137), serine glyoxylate aminotransferase (sS8_1474), hydroxypyruvate reductase (sS8_1475), D-glycerate-3-kinase (sS8_1476), enolase (sS8_1801), malate dehydrogenase (sS8_4317) and malyl-CoA lyase (sS8_2223) but lacks other genes. The *M*. *marinum* S8 genome contained all Calvin-Benson-Bassham cycle genes, such as ribulose-1,5- bisphosphate carboxylase (sS8_2432), phosphoglycerate kinase (sS8_4941), glyceraldehyde-3-phosphate dehydrogenase (sS8_1782), triosephosphate isomerase (sS8_0238), fructose-1,6-bisphosphate aldolase (sS8_1130), transketolase (sS8_1131), ribulose-phosphate 3-epimerase (sS8_ 2460) and phosphoribulokinase (sS8_4630). NAD-dependent FDH (*fdhA1*, *B1*, *C1*, *D1* and *E1*; sS8_0312–0316) and tungsten-containing FDH (*fdhA2* and *B2*–sS8_2524 and 2525) were also found. The genome was also found to contain genes for the TCA cycle.

### RNA-Seq of *M*. *caenitepidi* Gela4 in pure culture and co-culture with the methanotroph

**Fig 2:**
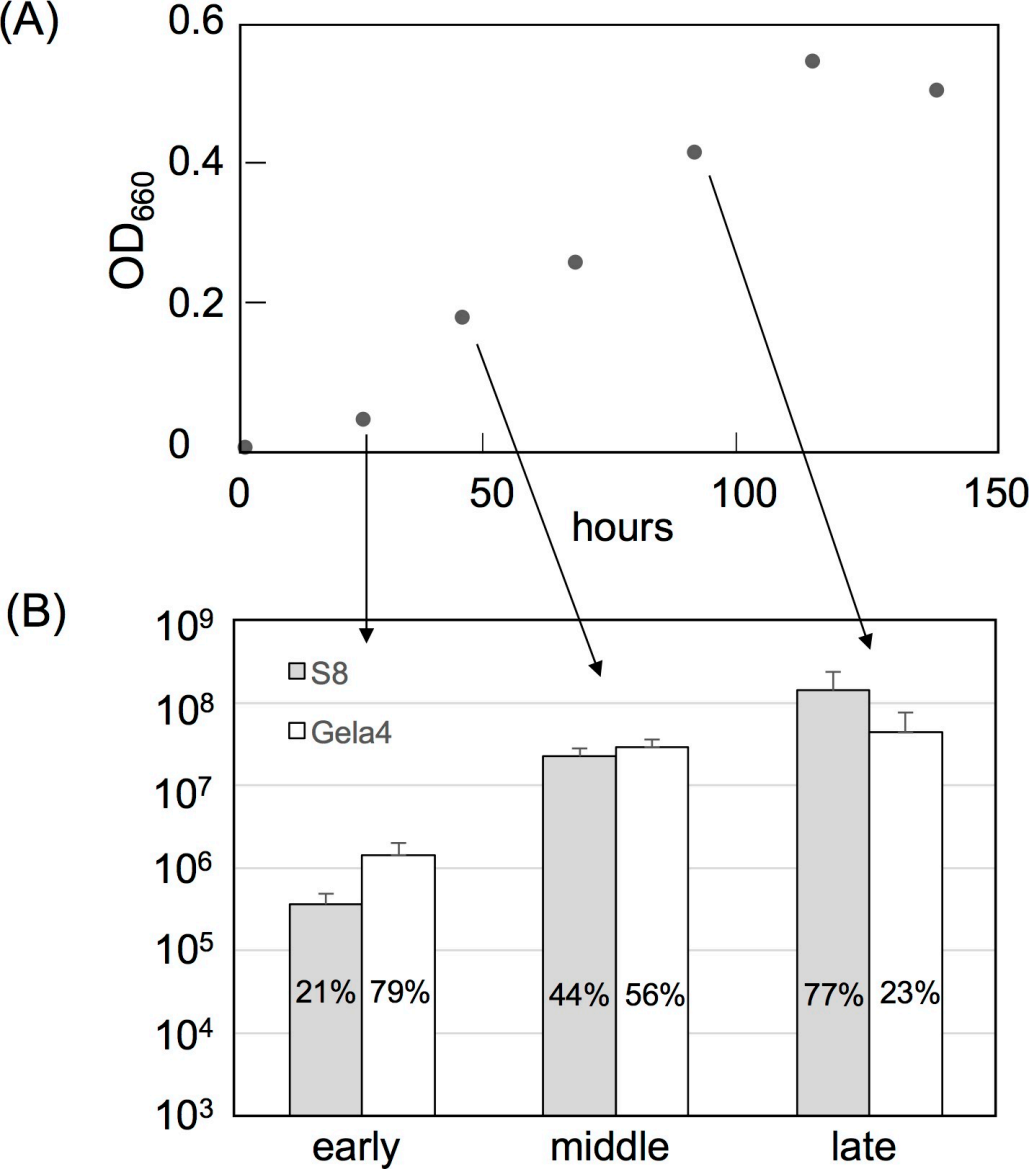
(A) Growth of the co-culture and (B) species composition at early, middle and late exponential growth phase. The gray bar and white bars represent number of *M*. *marinum* S8 cells and *M*. *caenitepidi* Gela4 cells, respectively. Error bars represent standard deviation of triplicates.

To reveal the effects of syntrophic growth with a methanotroph on the gene expression profiles of *M*. *caenitepidi* Gela4, RNA-Seq was conducted on *M*. *caenitepidi* Gela4 cells grown on methanol in pure culture and to those grown in a co-culture with the methanotroph *M*. *marinum* S8 in a medium supplemented with methane (S2 Fig). The specific growth rates of *M*. *caenitepidi* Gela4 in pure culture and the co-culture were 0.03 and 0.02 (h^−1^), respectively. Fig 2 presents (A) growth of the co-culture and (B) species composition at three different growth phases. Percentage of *M*. *marinum* S8 in co-culture increased from 21% to 77% during growth. The complete genome sequences of the two species enabled unambiguous separation of the sequenced reads between the two species in the co-culture samples (S3 and S4 Tables). When *M*. *caenitepidi* Gela4 was grown in co-culture with *M*. *marinum* S8, 24% of the uniquely mapped transcriptome data originated from *M*. *caenitepidi* Gela4 and 76% originated from *M*. *marinum* S8 on average. Pearson correlation coefficients of the expression levels between replicates were high in both pure culture (0.998) and co-culture (0.992) (S3 Fig), confirming high reproducibility of the RNA-Seq data. Full gene expression datasets for *M*. *caenitepidi* Gela4 and *M*. *marinum* S8 are presented in S5 Table and S6 Table, respectively.

**Fig 3:**
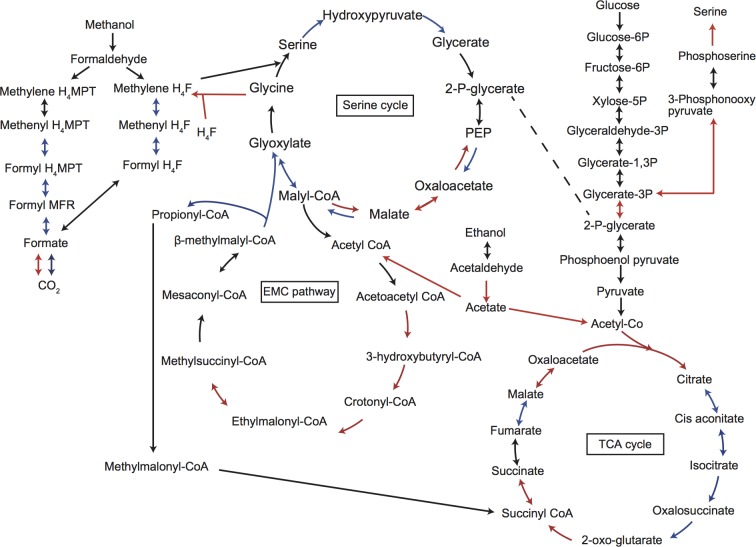
Predicted central carbon metabolic pathway of *M*. *caenitepidi* Gela4. Red and blue arrows indicate genes that were upregulated (log2FC > 0.3, FDR < 0.05) and downregulated (log2FC < −0.3, FDR < 0.05) in co-culture.

Expression of the central metabolism genes in *M*. *caenitepidi* Gela4 for both culture conditions, presented as RPKM are shown in Table 1. The expression of central metabolism genes in *M*. *marinum* S8 in co-culture with *M*. *caenitepidi* Gela4, presented as RPKM, is shown in S8 Table. Fig 3 shows the central carbon metabolism of *M*. *caenitepidi* Gela4 and genes that were significantly up- or downregulated.

**Table 1:** Expression profiles of major genes of the central metabolism of *M*. *caenitepidi* Gela4 cells grown on methanol in pure culture and in co-culture with *M*. *marinum* S8; log2FC refers to log-fold changes in expression, and FDR refers to false discovery rate.

	Predicted gene product	Gene	RPKM (pure culture)	RPKM (co-culture)	log2FC (co-culture/pure culture)	FDR
**Methanol oxidation**						
GL4_0421	Methanol dehydrogenase large subunit	*mxaF*	42779	40524	-0.08	0.308
GL4_0422	Methanol dehydrogenase small subunit	*mxaJ*	8017	8518	0.09	0.330
GL4_0423	Cytochrome c-L	*mxaG*	7201	6546	-0.14	0.074
GL4_0424	Methanol dehydrogenase, small subunit	*mxaI*	18826	21130	0.17	0.041
GL4_0437	Methanol dehydrogenase	*xoxF1*	1117	1368	0.29	0.001
GL4_1130	Methanol dehydrogenase	*xoxF2*	80	98	0.29	0.001
GL4_1360	Methanol dehydrogenase	*xoxF3*	1123	547	-1.04	0.000
GL4_1361	Methanol dehydrogenase	*xoxF4*	1918	1115	-0.78	0.000
**Serine cycle**						
GL4_1969	Serine hydroxymethyltransferase	*glyA*	523	571	0.13	0.097
GL4_3378	Serine-glyoxylate aminotransferase	*sga*	320	162	-0.98	0.000
GL4_2615	Serine-glyoxylate aminotransferase	*sga2*	1023	467	-1.13	0.000
GL4_3377	Hydroxypyruvate reductase	*hpr*	568	235	-1.27	0.000
GL4_3381	D-glycerate 2-kinase	*gckA*	78	89	0.18	0.021
GL4_2130	Enolase	*eno*	176	201	0.20	0.021
GL4_3372	Phosphoenolpyruvate carboxylase	*ppc*	248	153	-0.69	0.000
GL4_0105	Malate dehydrogenase	*mdh*	272	417	0.61	0.000
GL4_3373	Malate-CoA ligase alpha chain	*mtkB*	705	405	-0.80	0.000
GL4_3374	Malate-CoA ligase beta chain	*mtkA*	684	435	-0.65	0.000
GL4_3371	Malyl-CoA lyase	*mcl1*	971	638	-0.61	0.000
**Acetyl-CoA synthetase and EMC pathway**						
GL4_0367	Acetyl-coenzyme A synthetase	*acsA*	98	1181	3.57	0.000
GL4_0014	Acetyl-CoA acetyltransferase	*atoB*	749	812	0.11	0.200
GL4_0015	Acetoacetyl-CoA reductase	*phaB*	331	418	0.34	0.001
GL4_3328	3-hydroxybutyryl-CoA dehydrogenase	*hbdA*	371	517	0.48	0.000
GL4_3337	Crotonyl-CoA carboxylase/reductase	*ccr*	869	1097	0.34	0.000
GL4_3336	Methylsuccinyl-CoA dehydrogenase	*mcd*	283	293	0.05	0.593
GL4_2084	Methylmalonyl-CoA epimerase	*Mcee*	173	165	-0.06	0.430
GL4_3338	Ethylmalonyl-CoA mutase	*ecm*	161	218	0.43	0.000
GL4_0095	Mesaconyl-CoA hydratase	*mch*	188	181	-0.05	0.367
GL4_3371	malyl-CoA lyase	*mcl1*	971	638	-0.61	0.000
GL4_0468	L-malyl-CoA/beta-methylmalyl-CoA lyase	*mcl2*	50	237	2.22	0.000
**TCA cycle**						
GL4_2103	Citrate synthase	*citA*	250	403	0.69	0.000
GL4_0055	Aconitate hydratase	*acnA*	294	153	-0.94	0.000
GL4_1694	Isocitrate dehydrogenase	*icd*	346	185	-0.90	0.000
GL4_0108	2-oxoglutarate dehydrogenase E1 component	*sucA*	386	652	0.75	0.000
GL4_0109	2-oxoglutarate dehydrogenase E2 component	*sucB*	265	585	1.14	0.000
GL4_0107	Succinyl-CoA ligase alpha chain	*sucD*	132	700	2.40	0.000
GL4_0106	Succinyl-CoA ligase beta chain	*sucC*	217	673	1.63	0.000
GL4_0100	Succinate dehydrogenase flavoprotein subunit	*sdhA*	252	217	-0.22	0.004
GL4_0101	Succinate dehydrogenase iron-sulfur protein	*sdhB*	296	274	-0.11	0.091
GL4_0098	Succinate dehydrogenase cytochrome b-556 subunit	*sdhC*	677	779	0.20	0.009
GL4_0099	Succinate dehydrogenase hydrophobic membrane anchor protein	*sdhD*	140	136	-0.05	0.585
GL4_1597	Fumarate hydratase	*fum*	131	104	-0.33	0.000
GL4_0105	Malate dehydrogenase	*mdh*	272	417	0.61	0.000
**Ethanol oxidation**						
GL4_0818	Alcohol dehydrogenase	*adhA*	78	56	-0.49	0.000
GL4_0813	Aldehyde dehydrogenase	*aldA*	222	6354	4.83	0.000

**Fig 4:**
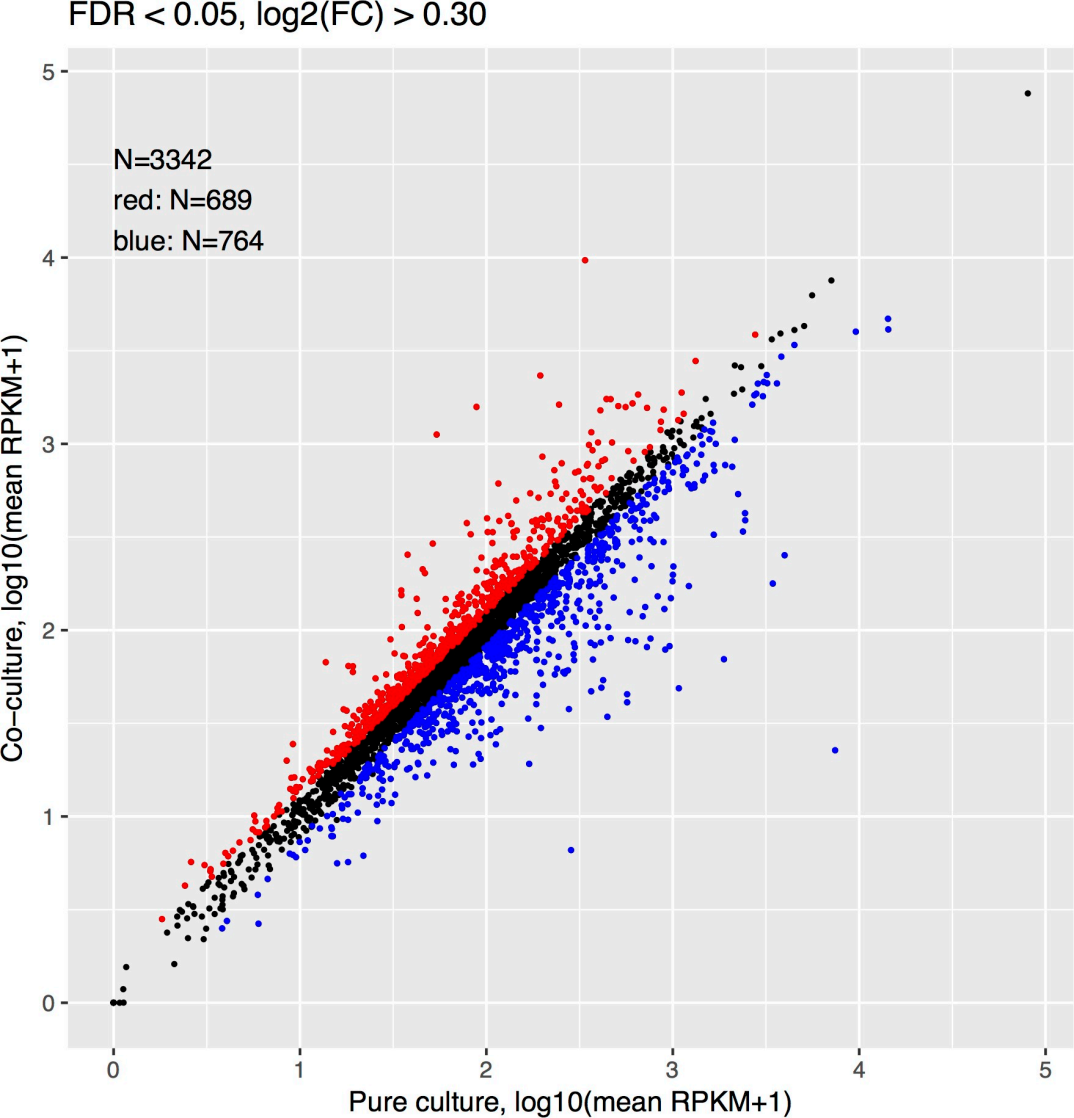
Differential expression of *M*. *caenitepidi* Gela4 genes in pure culture and co-culture. Red and blue points represent genes whose expression was upregulated (log2FC > 0.30, FDR < 0.05) and downregulated (log2FC < −0.30, FDR < 0.05), respectively.

**Fig 5:**
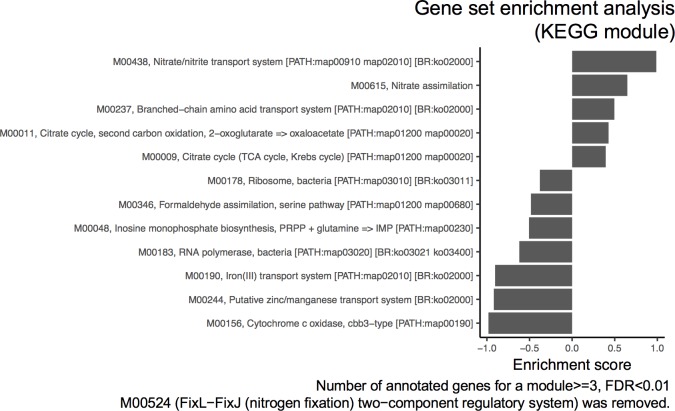
The results of gene set enrichment analysis on KEGG modules. The modules with FDR < 0.01 are shown according to the enrichment scores.

Differential gene expression analysis revealed that 689 genes of *M*. *caenitepidi* Gela4 were significantly upregulated [log2FC (fold-change) > 0.30, false discovery rate (FDR) < 0.05], and 764 genes were downregulated in co-culture when compared with pure culture (log2FC < −0.30, FDR < 0.05; Fig 4). To identify pathway modules enriched for differentially expressed genes, genes in *M*. *caenitepidi* Gela4 were assigned to KEGG pathway modules using metabolic and physiological potential evaluator [18], and gene set enrichment analysis (GSEA) [25] was performed using the pathway module annotations. Despite the low number of genes assigned to KEGG pathway modules (656 of 3,342 coding genes), 5 and 7 modules were significantly upregulated and downregulated, respectively (Fig 5, FDR < 0.01). Among the upregulated pathway modules were ‘Citrate cycle, second carbon oxidation, 2-oxoglutarate = > oxaloacetate’, and ‘Citrate cycle (TCA cycle, Krebs cycle)’, whereas among the downregulated modules were ‘Formaldehyde assimilation, serine pathway’ and ‘Cytochrome c oxidase, cbb3-type’. We also performed the hypergeometric test for association of KEGG pathway modules to up- or downregulated genes, which yielded similar results (S4 Fig, FDR < 0.05).

### Down-regulation of methanol assimilation pathways in *M*. *caenitepidi* Gela4 in co-culture

In *M*. *marinum* S8, genes involved in methane oxidization, including the methane monooxygenase (*pmo* operon) genes, methanol dehydrogenase (*mxaFJ*), hexulose-6-phosphate synthase, NAD-dependent formate dehydrogenase, and *fae*, showed high expression (S8 Table). This suggested that methane oxidization occurs in *M*. *marinum* S8 as expected during the co-culture.

**Fig 6:**
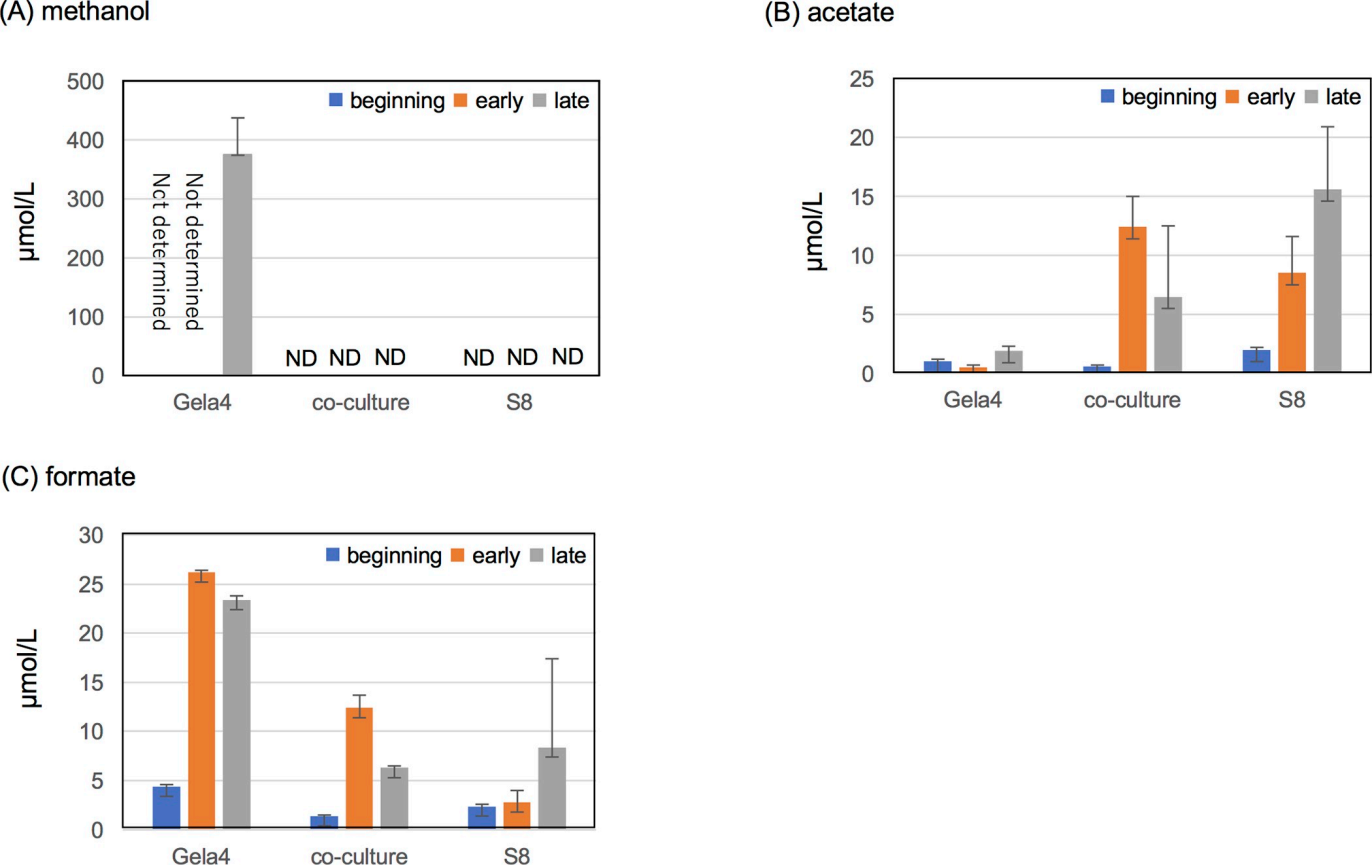
**Concentrations of (A) methanol, (B) acetate, and (C) formate in culture supernatants taken at the beginning of the incubation, and early and exponential phase of *M*. *caenitepidi* Gela4, *M*. *marinum* S8, and co-culture.** ND: not detected. Error bars represent standard deviation of triplicate experiments. Culture supernatants of *M*. *caenitepidi* Gela4 at the beginning of the incubation and early exponential phase was not analysed due to high concentration (1% in the liquid phase at the beginning) for the analysis.

In *M*. *caenitepidi* Gela4, genes encoding enzymes for the transformation of methanol to formaldehyde (*mxa* operon) and formaldehyde oxidation (*fae*) were among the most highly expressed in both pure culture and co-culture, and no significant changes in the expression levels of *mxaF* and *mxaJ* genes were observed (false discovery rate (FDR) > 0.05) (Table 1). This implied that methanol is a primary carbon source of the methylotroph in co-culture with the methanotroph. However, surprisingly, methanol was below the detection limit in both pure culture of the methanotroph or co-culture (Fig 6A). Considering that methanol dehydrogenase subunit 1 precursor is a major protein in both acetate-grown and methanol-grown *Methylobacter extorquens* AM1 [26], high expression of *mxa* operon genes and *fae* may not necessarily imply growth on methanol.

In line with undetectable concentration of methanol, many genes in pathways involved in carbon assimilation through formaldehyde (the serine pathway (*sga*, *hpr*, *ppc* and *mtk*), and dissimilation (H_4_MPT pathway and H_4_F pathway) were significantly downregulated in *M*. *caenitepidi* Gela4 grown in co-culture compared to that in pure culture (Fig 3). In addition, ‘Formaldehyde assimilation, serine pathway’ was among the downregulated KEGG modules (Fig 5). These results highlighted a downregulation of methanol utilization in *M*. *caenitepidi* Gela4 in co-culture compared to that in pure culture.

### Up-regulation of TCA cycle in *M*. *caenitepidi* Gela4 in co-culture

Next, we focussed on additional genes in central carbon metabolism pathways that were found to be upregulated in *M*. *caenitepidi* Gela4 in co-culture. The most highly upregulated gene was aldehyde dehydrogenase (*aldA*; GL4_0813; log2FC 4.83). The gene encoding the acetate oxidation enzyme, acetyl-CoA synthetase (*acsA*, GL4_0367), was also significantly upregulated (log2FC 3.57, Table 1), which would lead to increased acetyl-CoA synthesis. The gene encoding the proton-translocating pyrophosphatase (*hppa*) (GL4_1976), which is involved in the cleavage of pyrophosphate, the by-product of acetyl-CoA synthetase, was also upregulated.

Some genes in the EMC pathway, which converts acetyl-CoA to glyoxylate and succinyl-CoA, were upregulated in *M*. *caenitepidi* Gela4 in co-culture. Genes encoding acetoacetyl-CoA reductase (*phaB*; GL4_0015), 3-hydroxybutyryl-CoA dehydrogenase (*hbdA*; GL4_3328), crotonyl-CoA carboxylase/reductase (*ccr*; GL4_3337), ethylmalonyl-CoA mutase (*ecm*; GL4_3338) and l-malyl-CoA/beta-methylmalyl-CoA lyase (*mcl2*; GL4_0468) [27] were significantly upregulated (Fig 3, Table 1).

In the TCA cycle, genes for citrate synthase (*citA*, GL4_2103), malate dehydrogenase (*mdh*, GL4_0105), succinyl-CoA synthethase (*sucC* and *sucD*; GL4_0106 and GL4_0107) and 2-oxoglutarate dehydrogenase (sucA and sucB; GL4_0108 and GL4_0109) were upregulated (Fig 3, Table 1). The TCA cycle was also found to be upregulated according to the pathway enrichment analysis (Fig 5 and S4 Fig).

Importantly, many of the above genes that were differentially expressed in *M*. *caenitepidi* Gela4 were consistent with the expression profile of the facultative methylotroph *Methylobacterium extorquens* AM1 grown on acetate as compared with growth on methanol [26]. Specifically, upregulation was observed in the genes involved in the TCA cycle. Furthermore, genes encoding the proton-translocating pyrophosphatase (*hppa*) (GL4_1976) and the NAD(P) transhydrogenase (GL4_3073–3075), which is used to balance redox equivalents in the cell, were upregulated in both *M*. *extorquens* AM1 grown on acetate and *M*. *caenitepidi* Gela4 in co-culture. PEP carboxykinase (GL4_0586), which is operating in an opposing fashion to the serine cycle PEP carboxylase, was also upregulated in both cases. In addition, downregulation of NAD-dependent FDH (GL4_0089, 1367 and 1368) and upregulation of tungsten-containing FDH (GL4_3220 and 3221) was observed in both cases. Collectively, these results suggested that carbon assimilation via the EMC pathway and energy generation via the TCA cycle also occur in *M*. *caenitepidi* Gela4 co-cultured with *M*. *marinum* S8, consistent with observations using ^13^C steady-state metabolic flux analysis in *M*. *extorquens* AM1 grown on acetate [26].

### *M*. *marinum* S8 excretes acetate and formate

To search for possible compounds cross-fed by the methanotroph to complement our RNA-Seq analyses, we measured the concentrations of organic acids in the culture supernatants. Acetate was produced at <15.6 μmol/L in the pure culture of *M*. *marinum* S8, while that in the co-culture and *M*. *caenitepidi* Gela4 was <12.4 and <1.9 μmol/L, respectively (Fig 6B). Formate was detected at a concentration of <26.2, <8.4, and <12.4 μmol/L in culture supernatants of *M*. *caenitepidi* Gela4, *M*. *marinum* S8, and the co-culture (Fig 6C). Acetaldehyde was below the detection limit in most of the culture supernatants, but a trace amount (590 nmol/L) was detected in one of the triplicate samples from the late exponential phase of *M*. *marinum* S8. Propionate was below the detection limit in all of the culture supernatants.

### Quantitative analyses of *acsA*, *hpr*, and *aldA* genes

RNA-Seq analysis suggested that *M*. *caenitepidi* Gela4 utilizes compounds other than methanol, possibly acetate, in co-culture. To further investigate and to monitor if the metabolic pathway changes with growth phase, real-time quantitative reverse transcription PCR was performed for *acsA*, *hpr* of the serine pathway and *aldA* genes of *M*. *caenitepidi* Gela4 in co-culture at early and late exponential growth phases. Pure cultures of *M*. *caenitepidi* Gela4 grown with methanol and acetate were also analysed as references. Student t-test revealed that the ratio of *acsA* and *hpr* expression patterns in *M*. *caenitepidi* Gela4 were higher in cells grown with acetate and in cells in co-cultured cells at late exponential phase than in cells grown with methanol (p < 0.05) (Table 2). Similar results were obtained for the ratio of *aldA* and *hpr*.

**Table 2:** Relative expression levels of *acsA*, *hpr* and *aldA* genes of *M*. *caenitepidi* Gela4 grown with methanol, acetate and in co-culture.

	*acsA*/*hpr*	*aldA*/*hpr*
*M*. *caenitepidi* with methanol	0.2±0.1	0.2±0.0
*M*. *caenitepidi* with acetate	5.3±0.3	35.3±1.6
co-culture at early exponemtial phase	7.9±6.2	13.1±9.8
co-culture at late exponemtial phase	6.8±1.8	14.8±2.9

### Divergent response of *xoxF* genes in *M*. *caenitepidi* Gela4 in co-culture

The *M*. *caenitepidi* Gela4 genome contains four *xoxF* genes that encode methanol dehydrogenases. XoxF is a protein that has gained recent attention regarding methylotrophy [28], and shares about 50% amino acid identity with MxaF [29]. The protein has been shown to be involved in methanol oxidation [30] and regulation of methanol dehydrogenase genes [31]. Among the *xoxF* genes, the expression of GL4_1360 and 1361 was significantly downregulated, whereas the expression of GL4_0437 and GL4_1130 was upregulated with log2FC values of 0.29 in co-culture (S5 Fig, Table 1). These results highlighted the divergent response of *xoxF* genes in *M*. *caenitepidi* Gela4 upon co-culture; this was in contrast to *mxaF*, which remained highly expressed.

## Discussion

In the current study, we compared gene expression of the facultative methylotroph *M*. *caenitepidi* Gela4 grown in pure culture with methanol as the sole carbon source or co-cultured with the methanotroph *M*. *marinum* S8 with methane as the sole carbon source. The cross-feeding compound from methanotrophs to non-methanotrophic bacteria has been often considered as methanol excreted by methanotrophs. Krause et al. (2017) [8] has examined this interrelationship in detail. The authors conducted transcriptome analysis of two obligate methylotroph strains in co-culture with the methanotroph *Methylobacter tundripaludum* and demonstrated that the obligate methylotroph relies solely on methanol in co-culture with *M*. *tundripaludum*. In the current study, we examined for the first time the response of a facultative methylotroph, *M*. *caenitepidi* Gela4, which can assimilate a wide range of substrates in addition to methanol, such as acetate, ethanol, formate and succinate [11], grown in co-culture with a methanotroph by transcriptome analysis.

We discovered that the expression levels of nearly half of the genes of *M*. *caenitepidi* Gela4 were altered during co-culture (Fig 4, Table 1). Of note, genes for acetyl-CoA synthesis, the EMC pathway and TCA cycle were upregulated during co-culture cultures (Fig 3, Table 1). Considering that acetyl-CoA is a starting compound of the EMC pathway and TCA cycle, compounds that can be used for the acetyl-CoA synthesis are cross-fed from *M*. *marinum* S8 to *M*. *caenitepidi* Gela4.

One possible source of acetyl-CoA in co-culture is acetate. Indeed, expression of acetyl-coenzyme A synthetase increased with a log2FC value of 3.57. Some methanotrophs are known to produce organic acids, e.g. formate and acetate [10, 32]. For example, gammaproteobacterial methanotroph, *Methylomicrobium alcaliphilum*, has been shown to produce organic acids under oxygen-limited conditions [10]. The pure culture of *M*. *marinum* S8 produced up to 16 μmol/L of acetate during growth (Fig 4B). Pyruvate dehydrogenase (sS8_5306) and acetate kinase (sS8_2955) were found in the *M*. *marinum* S8 genome, suggesting that *M*. *marinum* S8 is also capable of ‘fermentation-based methanotrophy’ as *M*. *alcaliphilum*. These results suggested that acetate is a carbon source for *M*. *caenitepidi* Gela4 in co-culture. Increased relative expression level of *acsA* to *hpr* in *M*. *caenitepidi* Gela4 grown with acetate as well as in co-culture was confirmed by real-time PCR analysis (Table 2), supporting that *M*. *caenitepidi* Gela4 utilizes acetate in co-culture. Two genes (GL4_0878 and 2347) were found to encode proteins homologous to acetate transporter of *M*. *extorquens* (ActP) with 26–27% amino acid identities. However, there was no significant difference in the expression levels of these two genes between pure culture and co-culture conditions. Functional acetate transporter in *M*. *caenitepidi* Gela4 should be identified in future studies.

Another possible source of acetyl-CoA is acetaldehyde. Indeed, the most highly upregulated gene of *M*. *caenitepidi* Gela4 co-cultured with *M*. *marinum* S8 was that encoding the aldehyde dehydrogenase gene. However, relatively high expression of *aldA* was observed not only in co-culture but also in *M*. *caenitepidi* Gela4 grown with acetate (Table 2), suggesting that *aldA* is involved in acetate metabolism. Substrate specificity of *aldA* in *M*. *caenitepidi* Gela4 and its role in acetate metabolism should be investigated in future studies.

While formate was also produced in *M*. *marinum* S8 at 8 μmol/L, it was not considered as a cross-feeding compound as *M*. *caenitepidi* Gela4 produced a higher concentration of formate (23 μmol/L).

Our results demonstrated that methanol assimilation pathways were downregulated in *M*. *caenitepidi* Gela4 in co-culture conditions (Fig 3). It is possible that low concentrations with a high turnover would be sufficient to maintain cross-feeding. However, the concentration of methanol, both in pure culture of *M*. *marinum* S8 and co-culture, was below the detection limit (< 310 nmol/L), which is more than 1,000 times lower than the previous report on *M*. *tundripaludum* cross-feeding methanol (1.24 mmol/L) [8]. Surprisingly, the methanol concentration in the culture supernatant of *M*. *marinum* S8 was also much lower than the values reported previously for other methanotrophic species (0.01–1.00 mmol/L) [33]. Considering that a higher concentration of acetate (16 μmol/L) was produced by *M*. *marinum* S8, methanol may not be a primary carbon source for *M*. *caenitepidi* Gela4 in co-culture with *M*. *marinum* S8, if any, in contrast to the previous study on an obligate methylotroph [8].

*M*. *alcaliphilum* produces acetate under oxygen-limited conditions [10]. In this study, relative expression of *acsA* of *M*. *caenitepidi* Gela4 in co-culture was also high in the early exponential phase (Table 2), which is not considered to be an oxygen-limited condition, although the difference was not significant by t-test. Methanol was not detected, but 8.5 μmol/L of acetate was detected in early exponential phase of *M*. *marinum* S8, suggesting that *M*. *caenitepidi* Gela4 may utilize acetate not only in the late exponential phase. Further study is required to elucidate the mechanism of acetate production and effects of environmental conditions such as oxygen concentration on acetate excretion by *M*. *marinum* S8.

XoxF is a rare-earth element-dependent methanol dehydrogenase [34]. Although we did not supplement the growth medium with rare-earth elements (Inductively coupled plasma-mass spectrometry analysis confirmed that the concentration of La, Ce, Nd, and Pr in the growth medium was < 1 μg/kg), *M*. *caenitepidi* Gela4 expressed three *xoxF* genes at relatively high RPKMs, and their expression was altered in co-culture with the methanotroph (S5 Fig). This was in contrast to *mxaF* genes that remained highly expressed. The expression of *xoxF* genes of *Methylotenera mobilis* 13 was also altered in co-culture [8]. Although further studies are required to elucidate the functions of these genes, the results suggested that *xoxF* genes may be involved in the response to a co-culture conditions, such as, low methanol concentration.

It has long been considered that the cross-feeding compound between methanotrophic and non-methanotrophic bacteria is primarily methanol. Our results presented here suggest that this is not always the case. Organic acids such as acetate can also be an important cross-feeding compound depending on the species and perhaps on environmental conditions. *M*. *marinum* S8 and *M*. *caenitepidi* Gela4 is a good co-culture model to further study methanol-independent cross-feeding from methanotrophs to non-methanotrophs.

## Supporting information

S1 Fig Number of duplicated genes in the *M*. *marinum* S8 genome. (PDF)

S2 Fig Schematic showing the transcriptome experiment. (PDF)

S3 Fig Pairwise comparison of gene expression levels for *M*. *caenitepidi* Gela4 cells in pure culture and co-culture. (PDF)

S4 Fig The results of hypergeometric tests for overlaps of KEGG modules with (a) upregulated or (b) downregulated genes. The modules with FDR < 0.05 and > = 3 annotated M. caenitepidi Gela4 genes are shown according to the value of log10 (FDR).
(PDF)

S5 Fig Heat maps showing the expression levels of four *xoxF* genes of *M*. *caenitepidi* Gela4 in pure culture and co-culture with *M*. *marinum* S8. Scaled RPKM was calculated as a z-score of RPKM values for each gene per culture.
(PDF)

S1 Table KEGG pathway modules detected for *M*. *caenitepidi* Gela4. (XLSX)

S2 Table KEGG pathway modules detected for *M*. *marinum* S8. (XLSX)

S3 Table Evaluation of cross mapping between *M*. *caenitepidi* Gela4 and *M*. *marinum* S8. (XLSX)

S4 Table Summary of S3 Table. (XLSX)

S5 Table Transcriptomics data of *M*. *caenitepidi* Gela4 grown in pure culture with methanol as well as in co-culture with *M*. *marinum* and methane. (XLSX)

S6 Table Transcriptomics data of *M*. *marinum* S8 grown in co-culture with *M*. *caenitepidi* Gela4 and methane. (XLSX)

S7 Table Duplicated genes identified in the *M*. *caenitepidi* Gela4 genome (upper table) and *M*. *marinum* S8 genome (lower table) using 95% identity as a threshold. (XLSX)

S8 Table Expression profiles of major genes in the central metabolism of *M*. *marinum* S8 grown with *M*. *caenitepidi* Gela4. (XLSX)
