# Correction: Possible cross-feeding pathway of facultative methylotroph *Methyloceanibacter caenitepidi* Gela4 on methanotroph *Methylocaldum marinum* S8

**DOI:** 10.1371/journal.pone.0251538

**Published:** 2021-05-06

**Authors:** Mio Takeuchi, Haruka Ozaki, Satoshi Hiraoka, Yoichi Kamagata, Susumu Sakata, Hideyoshi Yoshioka, Wataru Iwasaki

There are several errors in S6 Table and S8 Table. Please view the correct S6 and S8 Tables below.

## Supporting information

S6 Table Transcriptomics data of *M. marinum* S8 grown in co-culture with *M. caenitepidi* Gela4 and methane. (XLSX)

S8 Table Expression profiles of major genes in the central metabolism of *M. marinum* S8 grown with *M. caenitepidi* Gela4. (XLSX)
