# Supplementary figures and images for: Possible cross-feeding pathway of facultative methylotroph Methyloceanibacter caenitepidi Gela4 on methanotroph Methylocaldum marinum S8
Source: PLoS One. 2019 Mar 14;14(3):e0213535. doi: 10.1371/journal.pone.0213535 (PMC6417678; doi:10.1371/journal.pone.0213535)

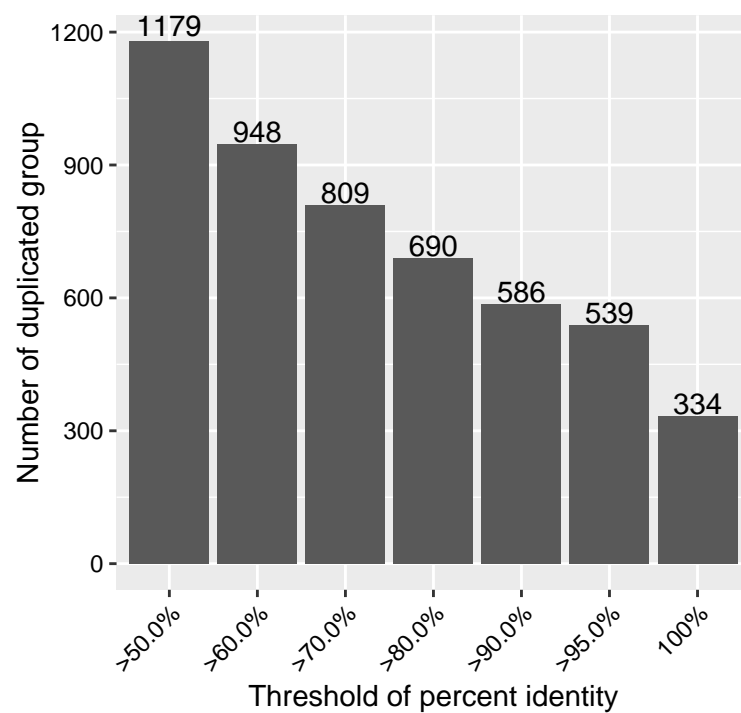

S1 Fig. Number of duplicated genes in the *M. marinum* S8 genome.

Supplement: S1 Fig — (PDF) [file pone.0213535.s001.pdf]

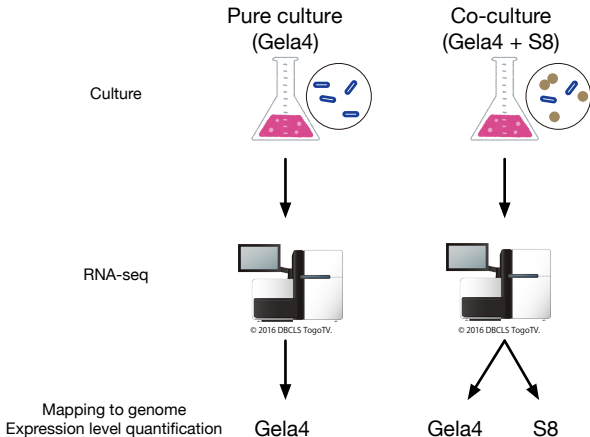

S2 Fig. Schematic showing the transcriptome experiment.

Supplement: S2 Fig — (PDF) [file pone.0213535.s002.pdf]
