# Supplementary material for: Possible cross-feeding pathway of facultative methylotroph Methyloceanibacter caenitepidi Gela4 on methanotroph Methylocaldum marinum S8
Source: PLoS One. 2019 Mar 14;14(3):e0213535. doi: 10.1371/journal.pone.0213535 (PMC6417678; doi:10.1371/journal.pone.0213535)
Supplement: S3 Fig — (PDF) [file pone.0213535.s003.pdf]

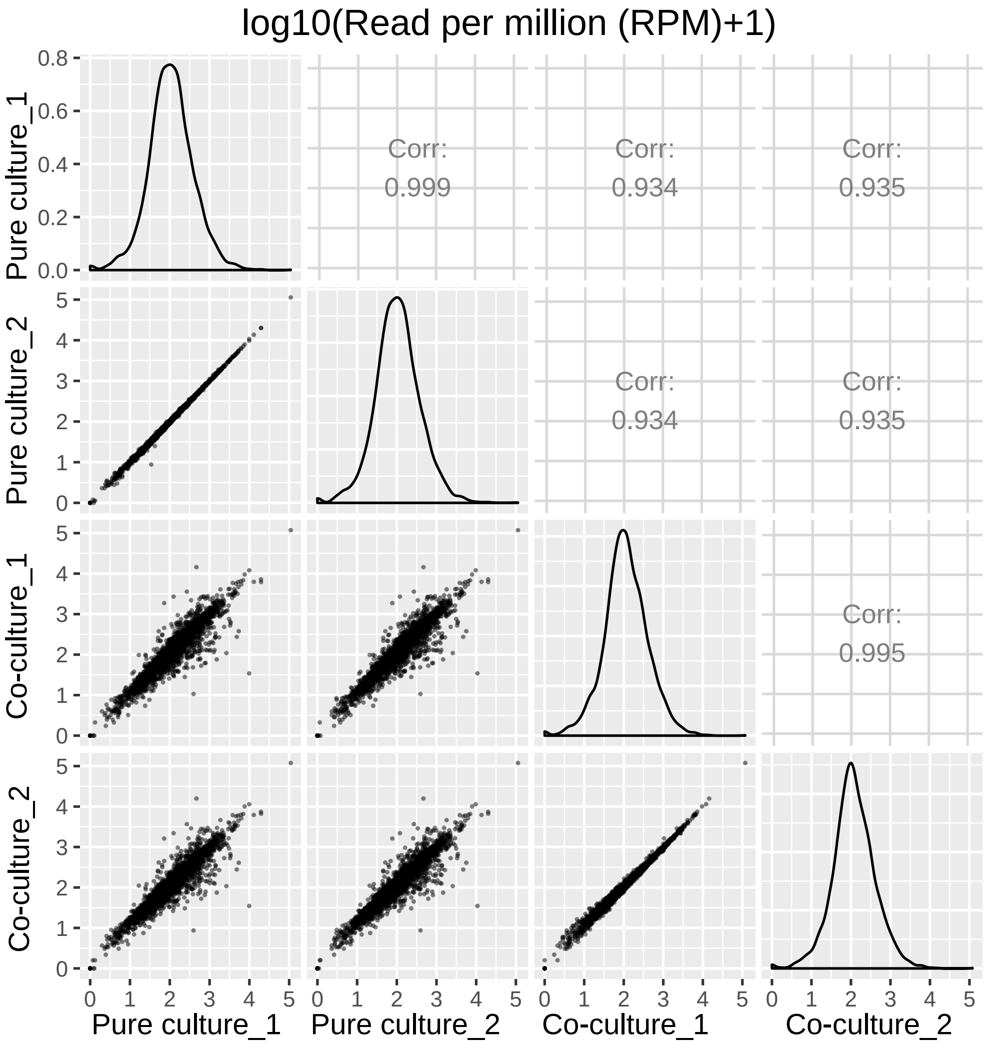

S3 Fig. Pairwise comparison of gene expression levels for *M. caenitepidi* Gela4 cells in pure culture and co-culture.
