# Supplementary material for: Possible cross-feeding pathway of facultative methylotroph Methyloceanibacter caenitepidi Gela4 on methanotroph Methylocaldum marinum S8
Source: PLoS One. 2019 Mar 14;14(3):e0213535. doi: 10.1371/journal.pone.0213535 (PMC6417678; doi:10.1371/journal.pone.0213535)
Supplement: S4 Fig — The results of hypergeometric tests for overlaps of KEGG modules with (a) upregulated or (b) downregulated genes. The modules with FDR < 0.05 and > = 3 annotated M. caenitepidi Gela4 genes are shown according to the value of log10 (FDR). (PDF) [file pone.0213535.s004.pdf]

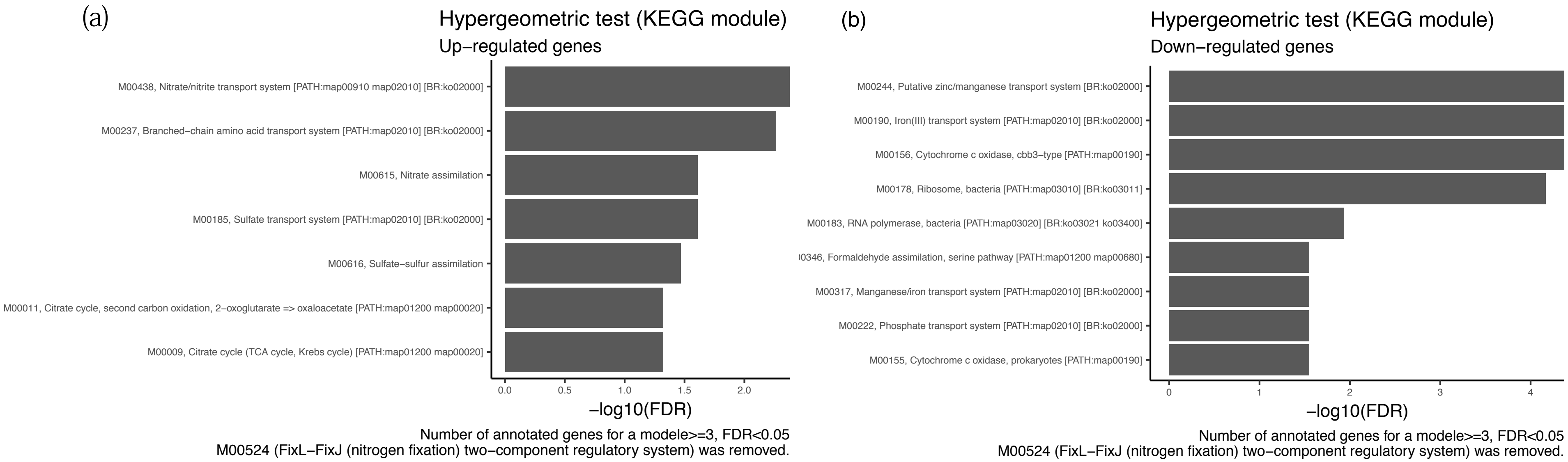

S4 Fig. The results of hypergeometric test for overlaps of KEGG modules with (a) up- or (b) downregulated genes.
The modules with FDR<0.05 and >=3 annotated *M. caenitepidi* Gela4 genes are shown according to the value of log10 (FDR).
