# Supplementary material for: Possible cross-feeding pathway of facultative methylotroph Methyloceanibacter caenitepidi Gela4 on methanotroph Methylocaldum marinum S8
Source: PLoS One. 2019 Mar 14;14(3):e0213535. doi: 10.1371/journal.pone.0213535 (PMC6417678; doi:10.1371/journal.pone.0213535)
Supplement: S5 Fig — Scaled RPKM was calculated as a z-score of RPKM values for each gene per culture. (PDF) [file pone.0213535.s005.pdf]

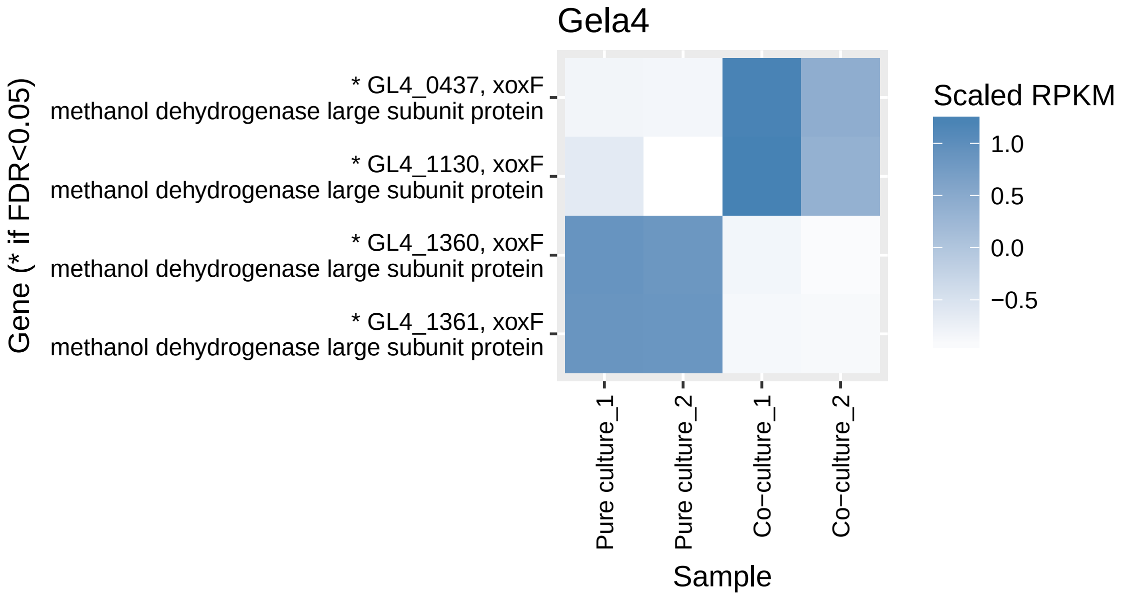

S5 Fig. Heat maps showing the expression levels of four xoxF genes of *M. caenitepidi* Gela4 in pure culture and co-culture with *M. marinum* S8. Scaled RPKM was calculated as a z-score of RPKM values for each gene per culture.
